# Eczema in early childhood is strongly associated with the development of asthma and rhinitis in a prospective cohort

**DOI:** 10.1186/1471-5945-12-11

**Published:** 2012-07-27

**Authors:** Laura B von Kobyletzki, Carl-Gustaf Bornehag, Mikael Hasselgren, Malin Larsson, Cecilia Boman Lindström, Åke Svensson

**Affiliations:** 1 Lund University, Institute of Clinical Research in Malmö, Skåne University Hospital, Department of Dermatology, Malmö, Sweden; 2 Karlstad University, Department of Public Health Sciences, Karlstad, Sweden; 3 Örebro University, School of Medicine, Örebro, Sweden; 4 County Council of Värmland, Primary Care Research Unit, Karlstad, Sweden

## Abstract

**Background:**

This study aimed to estimate the association between eczema in early childhood and the onset of asthma and rhinitis later in life in children.

**Methods:**

A total of 3,124 children aged 1–2 years were included in the Dampness in Building and Health (DBH) study in the year 2000, and followed up 5 years later by a parental questionnaire based on an International Study of Asthma and Allergies in Childhood protocol. The association between eczema in early childhood and the incidence of asthma and rhinitis later in life was estimated by univariable and multivariable logistic regression modelling.

**Results:**

The prevalence of eczema in children aged 1–2 years was 17.6% at baseline. Children with eczema had a 3-fold increased odds of developing asthma (adjusted odds ratio [aOR], 3.07; 95% confidence interval (CI) 1.79–5.27), and a nearly 3-fold increased odds of developing rhinitis (aOR, 2.63; 1.85–3.73) at follow-up compared with children without eczema, adjusted for age, sex, parental allergic disease, parental smoking, length of breastfeeding, site of living, polyvinylchloride flooring material, and concomitant allergic disease. When eczema was divided into subgroups, moderate to severe eczema (aOR, 3.56; 1.62–7.83 and aOR, 3.87; 2.37–6.33, respectively), early onset of eczema (aOR, 3.44; 1.94–6.09 and aOR, 4.05; 2.82–5.81; respectively), and persistence of eczema (aOR, 5.16; 2.62–10.18 and aOR, 4.00; 2.53–6.22, respectively) further increased the odds of developing asthma and rhinitis. Further independent risk factors increasing the odds of developing asthma were a parental history of allergic disease (aOR, 1.83; 1.29–2.60) and a period of breast feeding shorter than 6 months (aOR, 1.57; 1.03–2.39). The incidence of rhinitis was increased for parental history of allergic disease (aOR, 2.00; 1.59–2.51) and polyvinylchloride flooring (aOR, 1.60; 1.02–2.51).

**Conclusion:**

Eczema in infancy is associated with development of asthma and rhinitis during the following 5-year period, and eczema is one of the strongest risk factors. Early identification is valuable for prediction of the atopic march.

## Background

The prevalence of eczema has increased to levels of public health relevance in the Western world, especially in children [1]. As the most frequent inflammatory condition in childhood, eczema affects physiological and psychological wellbeing of affected children and results in substantial costs [2,3]. It has been suggested that early life eczema is a risk factor for the development of asthma later in life [4]. However, evidence for the progression to asthma comes mainly from cross-sectional studies [5]. There are only a few prospective cohort studies that have investigated the association between early life eczema and later onset of asthma and rhinitis [6-11]. Some of the existing longitudinal studies found no association between eczema and later onset of asthma [6-8], and other prospective studies found an eczema/asthma relationship much weaker than expected [9]. This overall weak association might be partly explained by a different effect across eczema subgroups on allergic airway diseases [12]. The severity of eczema has been found to be more closely associated with the risk of developing asthma than the timing of onset, or duration of eczema symptoms [12-14]. We examined whether eczema in early childhood predicts the later onset of asthma and rhinitis in children and determined the importance of severity, time of onset and persistency of the childhood eczema by analysing data from a large prospective population-based cohort with a follow-up period of 5 years.

## Methods

### Data collection

The methods of the population-based Swedish Dampness in Buildings and Health (DBH) study have been published by Bornehag et al.. and Larsson et al. [15,16].

The DBH study started in 2000, and a questionnaire based on an International Study of Asthma and Allergies in Childhood (ISAAC) protocol [17] was given to parents of all children aged 1 to 5 years living in the county of Värmland (DBH-I). Follow-up was performed in 2005 in children who were aged 1 to 3 years in 2000 (DBH-III). In this study we investigated the children initially aged 1–2 years.

Four thousand and twenty children initially aged 1–2 years had answered a baseline questionnaire in 2000 based on an ISAAC protocol. The cohort of the current study consisted of 3,124 children who responded both to the baseline and a follow-up questionnaire in 2005 (response rate: 77.7%).

Inclusion criteria of the baseline survey were all children living in Värmland aged 1–5 years whose parents gave consent to participate and who answered a postal questionnaire. The study was approved by the regional ethical committee in Örebro.

## Definition of variables

The original ISAAC questions were used. In addition, questions on doctor-diagnosed asthma and doctor-diagnosed rhinitis were included. The additional questions were: “Has your child been diagnosed with asthma by a physician?” and “Has your child been diagnosed with rhinitis by a physician?” The main outcomes were “5-year cumulative incidence of asthma” and “5-year cumulative incidence of rhinitis”. The “5-year cumulative incidence of asthma” was defined as no report of physician-diagnosed asthma and no wheezing at baseline in 2000, but physician-diagnosed asthma was reported in 2005. The “5-year cumulative incidence of rhinitis” was defined as no report of physician-diagnosed rhinitis in 2000, but physician-diagnosed rhinitis was reported in 2005.

The main explanatory variable was eczema. Eczema in early childhood was defined by affirmative responses to the question “Has your child had an itchy rash at any time in the last 12 months?” in the baseline questionnaire in 2000.

Secondary analysis was performed for subgroups of eczema, persistent eczema, severe eczema and early onset of eczema, and their association with later development of allergic airway diseases. Persistent eczema was assumed when children reported signs of eczema both at baseline and at follow-up; the questions asked were: “Has your child ever had an itchy rash (eczema) which was coming and going for at least 6 months” and “Has your child had this itchy rash at any time in the last 12 months?” in 2000, and “Has your child had this itchy rash at any time in the last 12 months?” in 2005. The onset of eczema was determined form the question “At what age did this itchy rash first occur?” in 2000, and it was categorised into “early onset” if symptoms occurred before the age of 1 year. The question “In the last 12 months, how often, on average, has your child been kept awake at night by eczema?” was included in the ISAAC eczema questionnaire as a measure of the severity of eczema, where sleep loss several times per week was considered to be severe eczema [17,18]. To simplify analysis, the severity of eczema was re-categorized, by using “any awakening at night” as an identifier for moderate to severe eczema, and “no awakening” was considered to be mild eczema. Information on single parenthood was obtained via the question “How many adults are currently living with the child”; two adults caring for the child could be any two adults.

### Statistical analysis

The association between eczema in early childhood and the development of asthma and rhinitis was estimated by using univariable logistic regression and expressed as odds ratios (ORs) with confidence intervals (CIs). Statistical significance was assessed using the likelihood ratio test (LRT; p < 0.05). Characteristics of participants and non-participants were compared in drop-out analysis using the chi-square test (p < 0.05). Crude analysis of the association between all other risk factors, and asthma and rhinitis were performed. Linear trends of variables with several categories were evaluated. In multivariable logistic regression analysis, the ORs were adjusted for sex, age of the child, socioeconomic variables (parental smoking, number of adults living with the child, and type of house), and potential confounding variables, which included all other factors (see Table 1). The potential confounding variables were added in descending order of strength of crude association (smallest p-value first). Factors still associated with asthma and rhinitis were kept in the model. The interaction between eczema and sex was explored. The effect of different subgroups of eczema on asthma and rhinitis was examined. Because of the strong association between persistence, early onset and severe eczema, three adjusted models for these groups are reported.

**Table 1:** Crude associations between risk factors at baseline and the incidence of asthma and rhinitis*

**Risk factor at baseline**	**Total number in category n (%)**	**Number with asthma n**	**Asthma OR (95% CI)**	**P-value**	**Number with rhinitis n**	**Rhinitis OR (95% CI)**	**P-value**
**Eczema**				<0.001			<0.001
No	2,573 (82.4)	101	1.0		107	1.0	
Yes	551 (17.6)	49	2.85 (1.72-4.71)		65	3.14 (2.27-4.36)	
**Persistence of eczema**							<0.001
No	2,886 (92.4)	58	1.0	<0.001	135	1.0	
Yes	238 (7.6)	15	4.20 (2.31-7.64)		37	3.92 (2.64-5.82)	
**Onset of eczema**							<0.001
No	2,483 (80.6)	46	1.0	<0.001	98	1.0	
Age ≥ one year	216 (6.9)	6	1.87 (0.7-4.45)		8	0.96 (0.46-2.00)	
Age < one year	386 (12.6)	21	3.63 (2.12-6.22)		66	5.02 (3.58-7.05)	
**Eczema severity**							<0.001
No	2,478 (79.47)	45	1.0	<0.001	98	1.0	
Mild	468 (15.0)	17	2.42 (1.36-4.29)		47	2.74 (1.90-3.95)	
Moderate to severe	173 (5.5)	11	4.55 (2.27-9.12)		27	4.74 (2.98-7.55)	
**Gender**							0.001
Female	1,546 (49.5)	25	1.0	0.002	65	1.0	
Male	1,579 (50.5)	48	2.16 (1.32-3.54)		107	1.67 (1.21-2.29)	
**Age of the child**							0.727
12-23 month	1,557 (49.8)	41	1.0	0.325	88	1.0	
24-35 month	1,568 (50.2)	32	0.79 (0.49-1.27)		84	0.95 (0.70-1.29)	
**Parental history of allergic disease**							<0.001
No parent	1,594 (51.0)	26	1.0	<0.001	47	1.0	
One parent	1,199 (38.4)	32	1.92 (1.14-3.25)		83	2.45 (1.69-3.53)	
Two parents	332 (10.6)	15 (7.2)	3.67 (1.90-7.08)		42	4.85 (3.12-7.55)	
**Asthma in the child**							<0.001
No	2,843 (91.4)	--	--		147	1.0	
Yes	269 (8.6)	--	--	--	25	4.10 (2.57-6.54)	
**Length of breastfeeding**							0.791
Longer than 6 month	2,043 (65.9)	44	1.0	0.021	112	1.0	
Up to 6 months	974 (31.4)	23	1.14 (0.68-1.90)		54	1.02 (0.73-1.43)	
No breastfeeding	82 (2.7)	6	3.32 (1.36-8.11)		6	1.35 (0.57-3.16)	
**Number of bedrooms with PVC**							0.116
No PVC in bedroom	1,300 (42.8)	23	1.0	0.184	60	1.0	
PVC in one bedroom	558 (18.4)	14	1.42 (0.72-2.79)		38	1.51 (0.99-2.30)	
PVC in two bedrooms	1,183 (38.9)	34	1.65 (0.96-2.82)		70	1.31 (0.92-1.87)	
**Type of building**							0.453
Single-family house	2,522 (82.4)	58	1.0	0.902	137	1.0	
Multi-family house	538 (17.6)	12	1.04 (0.55-1.96)		33	1.13 (0.75-1.70)	
**Parental smoking**							0.357
No	2,431 (77.8)	56	1.0	0.691	139	1.0	
Yes	694 (22.2)	17	1.12 (0.64-1.94)		33	0.83 (0.56-1.23)	
**Number of adults living with the child**							0.830
Two or more adults	2,853 (93.8)	66	1.0	0.320	157	1.0	
One adult	190 (6.2)	6	1.54 (0.65-3.62)		11	1.07 (0.57-2.01)	

3,124 children aged 1–2 in 2000 in Värmland, Sweden, were included in the analysis.

*during the 5-year follow-up period.

Clustering by families was considered. No clustering effects were found after statistical assessment. The results obtained using robust standard errors, generalised estimating equations, and random effect modelling were similar to those obtained from the analysis of single subjects in terms of the exposure outcome relationship, and no evidence for significant intra-cluster correlation was found. Therefore, ordinary logistic regression models are shown that ignore clustering. The Hosmer and Lemeshow goodness-of-fit test statistic provided evidence that the models were a good fit to the data [19]. We undertook a sensitivity analysis and used questions that asked about symptoms of asthma/rhinitis (wheezing and runny nose) and questions that asked about doctors’ diagnoses (asthma and rhinitis). This was performed in case people’s perceptions of symptoms are stronger indications of health than reports of doctors’ diagnoses.

### Power

Assuming that 7.3% of children with eczema and 2.5% of children without eczema develop asthma later in life, this study had the power to detect an odds ratio of 2.8 with 96% power.

All analyses were carried out using STATA, version 11 (STATA Corp., College Station, TX).

## Results

The study population consisted of 3,124 children, aged 1–2 years from 2,526 families due to siblinghood. The study population showed equal distribution regarding age and sex. Most children were breastfed (97.4%), and living with two adults (93.8%) in a single-family house (82.4%), with non-smoking parents (77.8%). A high proportion of children had at least one parent with a history of allergic disease (49.0%, Table 1). In the 1–2-year-old children, the prevalence of baseline eczema was 17.6% (n = 551; 95% CI, 16.3–19.0%). The cumulative 5-year incidence of asthma was 3.1 % (2.5–3.9) and rhinitis was 5.6% (4.8–6.5).

### Drop out in the study

Of 4,020 children aged 1–2 years, who participated in the baseline survey in 2000, 3,124 children participated in the follow-up in 2005. A total of 896 children did not continue participating in the study. There were no differences in background (age and sex) and health factors (prevalence of eczema, asthma, wheezing and rhinitis, and parents with history of allergic disease) measured at baseline between the children participating in both surveys compared with the “drop-outs” (p > 0.05). However, there was a higher prevalence of socioeconomic risk factors, parental smoking (30.5% vs. 22.2%; p < 0.001), one adult living with a child (11.1% vs. 6.2%; p < 0.001), and living in a multifamily house (27.0% vs. 17.6%; p < 0.001) in drop-outs compared with that in participants.

### Odds of developing asthma and rhinitis

Unadjusted analyses showed that children with eczema at baseline had more than a 2-fold increase in the odds of developing asthma (OR, 2.85; 95% CI, 1.72–4.71) and a 3-fold increase in the odds of developing rhinitis (OR, 3.14; 2.27–4.36) compared with children without eczema at baseline. The odds for developing asthma and rhinitis remained increased after adjustment (aOR, 3.07; 1.79–5.27 and aOR, 2.63; 1.85–3.73, respectively) for eczema, sex, age, family history of allergic disease, asthma, length of breastfeeding, PVC-flooring material in the home, type of building, environmental tobacco smoke, and number of adults living with the child (Table 2).

**Table 2:** Factors associated with the 5-year cumulative incidence of asthma and incidence of rhinitis in eczema patients aged 1–2 years in Sweden

**Risk factor at baseline**	**Incidence of asthma (N = 2063)**		**Incidence of rhinitis (N = 2751)**	
	Adjusted odds ratio* (95% confidence interval)	**P-value**	Adjusted odds ratio* (95% confidence interval)	**P-value**
*Eczema at baseline*				<0.001
No	1.0	<0.001	1.0	
Yes	3.07 (1.79-5.27)		2.63 (1.85-3.73)	
*Gender*				0.006
Female	1.0	0.003	1.0	
Male	2.20 (1.30-3.72)		1.59 (1.14- 2.23)	
*Age of the child*				0.290
12-23 month	1.0	0.340	1.0	
24-35 month	0.78 (0.47-1.30)		0.84 (0.60-1.16)	
*Parental history of allergic disease*^*&*^				<0.001
No parent with allergic disease	1.0	<0.001	1.0	
Parent(s) with history of allergic disease	1.83 (1.29-2.60)		2.00 (1.59-2.51)	
*Asthma in the child*				<0.001
No	--	--	1.0	
Yes			2.80 (1.66-4.70)	
*Breastfeeding*^*&&*^				0.446
Breastfeeding longer than 6 month	1.0	0.044	1.0	
Breastfeeding up to 6 month	1.57 (1.03-2.39)		1.13 (0.83-1.54)	
*Number of bedrooms with PVC*				0.106
No PVC in bedroom	1.0	0.288	1.0	
PVC in one bedroom	1.36 (0.65-2.83)		1.60 (1.02- 2.51)	
PVC in two bedrooms	1.57 (0.89-2.78)		1.30 (0.90-1.90)	
*Type of dwelling*				0.546
Single family house	1.0	0.605	1.0	
Multi family house	1.21 (0.60-2.44)		1.15 (0.73-1.81)	
*Smoking by parents*				0.707
No	1.0	0.810	1.0	
Yes	0.92 (0.47-1.79)		0.92 (0.60-1.42)	
*Number of adults living with the child*				0.942
Two or more	1.0	0.208	1.0	
One	0.43 (0.10-1.81)		1.03 (0.50-2.11)	

*Adjusted by logistic regression for all other variables listed in the table.

^&^linear effect aOR, with reference category no parent; other categories one parent, and two parents.

^&&^ linear effect aOR, with reference category longer than 6 months; other categories up to 6 months, and no breastfeeding.

### Different subgroups of children with eczema

In adjusted analysis, children with persistence of eczema, early onset of eczema, or moderate to severe eczema had even higher odds of developing asthma and rhinitis than children with eczema in general. The odds of developing asthma and rhinitis was 5-fold in children with persistence of eczema compared with the absence of eczema (aOR, 5.16; 2.62–10.18 and aOR, 4.00; 2.53–6.22, respectively). Early onset of eczema was a strong risk factor for the incidence of asthma (aOR, 3.44; 95% CI, 1.94–6.09) and the incidence of rhinitis (aOR, 4.05; 95% CI, 2.82–5.81) compared with children without eczema, whereas there was no significant association between the late onset of eczema and the incidence of asthma (aOR, 2.07; 0.78–5.49) and rhinitis (aOR, 0.96; 0.45–2.03). Both mild and moderate to severe eczema were associated with the incidence of asthma and rhinitis compared with children without eczema, with a higher odds of developing asthma and rhinitis in moderate to severe eczema (aOR, 3.56; 1.62–7.83 and aOR, 3.87; 2.37–6.33, respectively) than mild eczema compared with children without eczema (aOR, 2.85; 1.57–5.19 and aOR, 2.37; 1.60–3.51, respectively) (Table 3).

**Table 3:** **Subgroups of eczema in children and the 5-year cumulative incidence of asthma and rhinitis**^**$**^

**Eczema group**	**Crude OR (95% CI)**	**P-value**	**Adjusted OR**^**#**^**(95% CI)**	**P-value**
	***Incidence of asthma****(N = 2063)*			
Duration of eczema				
Never eczema	1.0	<0.001	1.0	<0.001
Eczema only in 2000	1.94 (0.93-4.06)		2.17 (1.01-4.65)	
Eczema only in 2005	1.97 (0.94- 4.13)		1.77 (0.77-4.11)	
Persistence of eczema^##^	4.95 (2.65-9.25)		5.16 (2.62-10.18)	
Onset of eczema				
Late onset of eczema^###^	1.87 (0.78-4.45)	<0.001	2.07 (0.78-5.49)	<0.001
Early onset of eczema ^###^	3.63 (2.12-6.22)		3.44 (1.94-6.09)	
Severity of eczema				
Mild eczema^###^	2.42 (1.36-4.29)	<0.001	2.85 (1.57-5.19)	<0.001
Moderate to severe eczema ^###^	4.55 (2.27-9.11)		3.56 (1.62-7.83)	
	***Incidence of rhinitis****(N = 2751)*			
Duration of eczema				
No eczema	1.0	<0.001	1.0	<0.001
Eczema only in 2000	2.52 (1.61-3.94)		2.25 (1.40-3.61)	
Eczema only in 2005	1.96 (1.20-3.21)		2.00 (1.19-3.37)	
Persistence of eczema^##^	4.91 (3.23-7.46)		4.00 (2.53-6.22)	
Onset of eczema				
Late onset of eczema^###^	0.96 (0.46-2.00)	<0.001	0.96 (0.45-2.03)	<0.001
Early onset of eczema ^###^	5.02 (3.58-7.05)		4.05 (2.82-5.81)	
Severity of eczema				
Mild eczema^###^	2.74 (1.90-3.95)	<0.001	2.37 (1.60-3.51)	<0.001
Moderate to severe eczema^###^	4.74 (2.98-7.55)		3.87 (2.37-6.33)	

$ If not otherwise stated, the comparisons are dichotomous with the referent category being the low-odds group (aOR = 1), and the high-odds group data shown in the table.

#Adjustments were made for gender, age, family history of allergic disease, asthma, length of breastfeeding, PVC-flooring material in the home, type of building, environmental tobacco smoke, and number of adults living with the child.

## Defined as eczema ever for at least 6 months, and current eczema in both 2000 and 2005.

### no eczema in 2000 as reference.

### Other risk factors for development of asthma and rhinitis during a 5-year period

In adjusted analysis, a parental history of allergic disease increased the odds of developing asthma and rhinitis by 2-fold. The odds of developing asthma was 2-fold and that of developing rhinitis was 59% higher in boys compared with girls. Further, asthma increased the odds of developing rhinitis by nearly 3-fold, and a period of breastfeeding shorter than 6 months increased the risk of developing asthma by 57% (Table 2). PVC-flooring material in the bedroom increased the incidence of asthma and rhinitis, but statistical significance was only reached for the relationship between PVC flooring-material and rhinitis. Interestingly, PVC-flooring material had a higher effect on girls for the incidence of asthma and rhinitis in all models (data not shown). Interactions between eczema and sex were explored and there was no effect of eczema on the incidence of asthma and rhinitis across sex strata (interaction p-values: 0.747 for asthma and 0.664 for rhinitis).

### Sensitivity analysis

For assessing the relationships between asthma and rhinitis and remission, we used different assessments of asthma and rhinitis to ensure that the relationship was stable and not dependent on which questions were used. We used both reports of symptoms and reports of doctors’ diagnoses. Both of these reports were similar in both crude and adjusted analysis; eczema increased the odds of developing rhinitis symptoms (OR, 2.81; 2.23–3.55 and aOR, 2.39; 1.86–3.08) and wheezing symptoms (OR, 2.10; 1.37–3.23 and aOR, 1.97; 1.24–3.12).

## Discussion

Our study found that eczema in early childhood was strongly associated with the development of asthma and rhinitis during the following 5-year period. Eczema was one of the strongest independent risk factors. Interestingly, when eczema was divided into subgroups, children with early onset of eczema, moderate to severe eczema, and persistence of eczema had the highest odds of developing asthma and rhinitis.

This population-based prospective study confirms that early eczema affects later development of asthma and rhinitis.

Although some previous prospective studies were not able to show an association between early childhood eczema and later development of asthma and rhinitis [6,8], our findings are robust and in line with the study by Arshad et al [9]. In addition, similar results regarding severity have been found in both Gustavsson’s and Ricci’s eczema cohorts [12,13], which reported that eczematous children with high severity scores were at increased risk of developing asthma. To the best of our knowledge, our study is the first prospective cohort to show that early onset of eczema or persistent eczema increases the odds of later onset of asthma in both boys and girls, which is in contrast to a previous study that only showed a relationship in boys [20]. Definitions of asthma and rhinitis are important for the interpretation of results [15]. Our sensitivity analysis confirmed that the association between eczema and asthma/rhinitis remained when symptom-based criteria for asthma and rhinitis were used.

### Possible explanations for the relationship between eczema and asthma and rhinitis

Evidence from several experimental studies has suggested that impaired epithelial function results in increased sensitization and IgE production [21]. In humans, the theory of epicutaneous sensitization is supported by the observation that exposing atopic children to topical emollients containing peanut protein leads to an increased risk of airway peanut sensitization [22]. Genetic factors, such as the common loss of function mutations within the filaggrin gene, are a risk factor for incident eczema and account for skin barrier dysfunction [23,24]. Recently, it has been shown that filaggrin mutations affect asthma, which supports the hypothesis that impaired skin function acts as a gateway for allergens, increasing the risk of atopic airways diseases [25].

### Advantages and limitations

A major advantage of our study was its prospective design, which made results less subject to recall bias and allowed assessment of temporal relationships. Our study had a large sample size compared with earlier studies [6,8]. Our results should be less prone to selection and ascertainment bias because of its population-based design. We also had a high response rate and limited loss to follow-up. There were no differences between the analysed sample and drop-outs in health-related variables; therefore results might have been biased towards 1. A higher prevalence of socioeconomic risk factors in children leaving the study, while assuming that low socioeconomic status is a risk factor for the incidence of asthma, might have biased the results towards 1 as well. Therefore, our conclusions that eczema is associated with later onset of asthma and rhinitis would not change if all children had participated. The advantages of the study design and performance allow generalization of results.

Reporting of eczema by questionnaire might have advantages compared with assessment by physicians, because eczema can be intermittent [26]. The term “*itchy flexural rash in the last 12 months”* has been shown to correlate well with diagnosis by a physician in a validation study performed in the UK on children aged 3–11 years [18]. Sensitivity in this previous study was 84% and specificity was 93% [18]. In addition, the ISAAC eczema questionnaire has been used for studies on pre-school children [26]. Although questions for diagnosis have been validated for eczema in 4–11-year-olds and for asthma in children aged 1–6 years [27], the ISAAC questionnaire has not been validated for parental information regarding preschool children. However, we consider that it is unlikely that there was differential misclassification; random misclassification would mean that any bias in our estimates was towards null.

Currently, there is no clear definition of persistence of eczema, which is consistent as reported by Williams [28]. Illi [7] and Möhrenschlager [29] considered eczema as persistent when signs of the disease were present at different points of time. In our study, persistence of eczema was defined as having had eczema at least three times. Therefore, the risk of including children with “short-term rashes only” might be low, but we cannot exclude the possibility that some children classified as having persistent eczema had longer symptom-free intervals. It would have been advantageous to assess the prevalence of eczema more often during the study period.

### Implications

Based on the results of our study, further evidence is required to unravel the underlying mechanisms of eczema in early childhood leading to asthma. Although we confirmed a relationship between childhood eczema and incident asthma and rhinitis, the association between childhood eczema and later asthma and rhinitis might still be separate and sequential, but are otherwise unrelated in the background of an atopic phenotype. Because the relationship between childhood eczema and incident asthma and rhinitis was temporal, strong, and linear, our findings suggest that the relationship might be causal. In this regard, impaired skin function might hypothetically be one explanatory factor [21], and the effect of early and successful treatment of eczema should be explored regarding the incidence of asthma as one outcome measure [30,31]. Further, it might be beneficial to estimate if objective measured sensitization (IgE) modifies or confounds the eczema/asthma relationship in future studies. Our study results have important implications for patient management since asthma can lead to high impairment and costs.

## Conclusions

Eczema in infancy independently increases the odds of developing asthma and rhinitis during the following 5-year period. This association is present for children with eczema at baseline; however when they are divided into subgroups (severe eczema, early onset of eczema and persistence of eczema) the odds of the incidence of asthma and rhinitis further increases. Identifying risk groups is important for healthcare planning.

## Abbreviations

DBH: Dampness in Building and Health; ISAAC: International Study of Asthma and Allergies in Childhood; PVC: Polyvinyl chloride.

## Competing interests

There is no conflict of interest among the authors Laura B. von Kobyletzki, Carl-Gustaf Bornehag, Mikael Hasselgren, Malin Larsson, Cecilia Boman Lindström, and Åke Svensson, between reporting of the study findings, and financial or non-financial interests that might bias the report.

## Authors’ contributions

LVK, AS and CGB take responsibility for the integrity of the data. LVK performed data analysis. LVK, AS, and CGB participated in the concept and design of the study. CGB, MH, ML, LVK, and CBL were involved in acquisition of data. LVK, AS, CGB, and ML interpreted the data. LVK drafted the manuscript. AS, CGB, MH, ML, and CBL critically revised the manuscript for important intellectual content. AS and CGB supervised the study. All authors read and approved the final manuscript.

## Pre-publication history

The pre-publication history for this paper can be accessed here:

http://www.biomedcentral.com/1471-5945/12/11/prepub
